# Occupational Exposure to Trichloramine and Trihalomethanes in Swedish Indoor Swimming Pools: Evaluation of Personal and Stationary Monitoring

**DOI:** 10.1093/annhyg/mev045

**Published:** 2015-07-07

**Authors:** Jessica Westerlund, Pål Graff, Ing-Liss Bryngelsson, Håkan Westberg, Kåre Eriksson, Håkan Löfstedt

**Affiliations:** ^1.^Department of Occupational and Environmental Medicine, Faculty of Medicine and Health, Örebro University, SE-701 85 Örebro, Sweden;; ^2.^Department of Clinical Medicine, School of Health Sciences, Örebro University, SE-701 85 Örebro, Sweden;; ^3.^Man-Technology-Environment Research Centre (MTM), Department of Science, Örebro University, SE-701 85 Örebro, Sweden;; ^4.^Department of Public Health and Clinical Medicine, Occupational and Environmental Medicine, Umeå University, SE-90187 Umeå, Sweden

**Keywords:** exposure assessment, exposure assessment methodology, trichloramine, trihalomethanes

## Abstract

**Introduction:**

Chlorination is a method commonly used to keep indoor swimming pool water free from pathogens. However, chlorination of swimming pools produces several potentially hazardous by-products as the chlorine reacts with nitrogen containing organic matter. Up till now, exposure assessments in indoor swimming pools have relied on stationary measurements at the poolside, used as a proxy for personal exposure. However, measurements at fixed locations are known to differ from personal exposure.

**Methods:**

Eight public swimming pool facilities in four Swedish cities were included in this survey. Personal and stationary sampling was performed during day or evening shift. Samplers were placed at different fixed positions around the pool facilities, at ~1.5 m above the floor level and 0–1 m from the poolside. In total, 52 personal and 110 stationary samples of trichloramine and 51 personal and 109 stationary samples of trihalomethanes, were collected.

**Results:**

The average concentration of trichloramine for personal sampling was 71 µg m^−3^, ranging from 1 to 240 µg m^−3^ and for stationary samples 179 µg m^−3^, ranging from 1 to 640 µg m^−3^. The air concentrations of chloroform were well below the occupational exposure limit (OEL). For the linear regression analysis and prediction of personal exposure to trichloramine from stationary sampling, only data from personal that spent >50% of their workday in the pool area were included. The linear regression analysis showed a correlation coefficient (*r* ^2^) of 0.693 and a significant regression coefficient *β* of 0.621; (95% CI = 0.329–0.912, *P* = 0.001).

**Conclusion:**

The trichloramine exposure levels determined in this study were well below the recommended air concentration level of 500 µg m^−3^; a WHO reference value based on stationary sampling. Our regression data suggest a relation between personal exposure and area sampling of 1:2, implying an OEL of 250 µg m^−3^ based on personal sampling.

## INTRODUCTION

Chlorination is a method that is commonly used to keep indoor swimming pool water free from pathogens. Chlorine is normally introduced as a salt, sodium hypochlorite (NaOCl) or calcium hypochlorite (Ca(OCl)_2_) (WHO, 2006). However, chlorination of swimming pools produces by-products (mono-, di-, and trichloramines) as the chlorine will react with nitrogen containing organic matter such as urea, sweat, dandruff, and skin flakes (Hailin *et al.*, 1990; Hery *et al.*, 1995). Trichloramine is the most volatile chloramine that is formed in this reaction and the most likely to be found in swimming pool atmospheres (Holzwarth *et al.*, 1984), the major route of exposure being inhalation.

Up till now, exposure assessments in indoor swimming pools have relied on stationary measurements at the poolside, used as a proxy for personal exposure. No occupational exposure limit (OEL) or threshold limit value (TLV) has been established for trichloramine, but the World Health Organization (WHO, 2006) has a recommended reference value for trichloramine of 500 µg m^−3^, determined as stationary sampling at the pool side. Earlier surveys in indoor swimming pools in France and Great Britain determined trichloramine concentrations between 100–570 and 140–600 µg m^−3^_,_ respectively (Massin *et al.*, 1998; Thickett *et al.*, 2002). Some later studies determined trichloramine concentrations between 40 and 520 µg m^−3^ (Jacobs *et al.*, 2007; Parrat *et al.*, 2012; Fornander *et al.*, 2013). Adverse health effects such as symptoms of the upper airways, nausea, and occular irritation have been reported among swimming pool employees (Hery *et al.*, 1995; Massin *et al.*, 1998; Thickett *et al.*, 2002; Jacobs *et al.*, 2007; Parrat *et al.*, 2012). The symptoms were particularly pronounced in those suffering from asthma (Carbonnelle *et al.*, 2002; Thickett *et al.*, 2002; Bernard *et al.*, 2003). Trichloramine levels between 100 and 570 µg m^−3^ showed increased prevalence of asthma in swimming pool staff (Thickett *et al.*, 2002) and a more recent study from Switzerland determined irritative symptoms among swimming pool employees at trichloramine levels of 200–300 µg m^−3^ (Parrat *et al.*, 2012).

Trihalomethanes (THMs) are also formed when chlorine reacts with organic materials in the pool water (Lahl *et al.*, 1981). The most common THMs in swimming pool atmospheres are chloroform (CHCl_3_), bromodichloromethane (CHCl_2_Br), dibromochloromethane (CHClBr_2_), and bromoform (CHBr_3_) (Lahl *et al.*, 1981; Aggazzotti *et al.*, 1998; Fantuzzi *et al.*, 2001). For the THMs, the main route of exposure is inhalation but dermal uptake should also be considered (Erdinger *et al.*, 2004).

CHCl_3_ and CHCl_2_Br have been classified by the International Agency for Research on Cancer (IARC) as possibly carcinogenic to humans (group 2B), (WHO-IARC, 1999). The Swedish OEL for CHCl_3_ is 10mg m^−3^ and the corresponding ACGIH TLV is 50mg m^−3^ (SWEA, 2005; ACGIH, 2006).

This exposure survey was part of a study on respiratory effects and symptoms among swimming pool workers to be presented elsewhere. In this study, we have determined the workers exposures to trichloramine and THMs by personal exposure measurements as well as the standard stationary sampling in Swedish indoor pool facilities. The use of stationary sampling as a basis for comparison with recommended WHO reference limits implied a special interest between exposure and stationary sampling in the swimming pool settings.

## METHODS

### Study object

Eight public indoor swimming pool facilities in four Swedish cities were included in our survey carried out during 2007–2009 winter seasons. The facilities consisted of 1–3 pools and the number of visitors per year varied between 18000 and 300000. All swimming pools were disinfected during recycling of the pool water using NaClO and filtration, sometimes in combination with ultraviolet (UV) light. The number of employees varied between 4 and 20 depending on facility size and opening hours. All facilities offered recreational swimming and swimming lessons; a few offered aquaerobics, baby swimming and training for swimming clubs. One facility also showed leisure pool activities. Characteristics of each facility are presented in Table 1.

**Table 1.** Characteristics of the eight public indoor swimming pool facilities investigated in this survey.

Pool facility	Employees (*n*)	Visitors/year	Type of pool	Pool size (m)	Water temp. (°C)	Disinfection method	Ventilation	Height of ceiling (m)
1	6	80000	Exercise pool	16×8	30	NaOCl	HRV	3
2	9	200000	Exercise pool	25×8	28	NaOCl	HRV	3
			Training pool	12×8	32			
			Whirlpool		34			
3	7	170000	Exercise pool	25×12	30	NaOCl, UV-light	HRV	3
			Whirlpool		35			
4	35 (rotation 2 facilities)	265000	Exercise pool	25×14	29	NaOCl	HRV	Exercise pool: 11
			Training pool	11×6	33			Training pool: 4
5		300000	Exercise pool	25×50	29	NaOCl, UV-light	HRV	6
			Training pool	12×6	33			
6	20	200000	Exercise pool	25×16	27	NaOCl	HRV	3
			Training pool	ø 12	32			Diving tower: 8
7	4	18000	Exercise pool	25×8	29	NaOCl	HRV	6
8	13	220000	Exercise pool	25×12	27	NaOCl	HRV	Exercise pool: 10
			Training pool	12×5	34			Leisure pool: 6
			Leisure pool	15×20	29			
			Children’s pool	3×4	37			
			Whirlpool		37			

HRV, heat recovery ventilation systems.

### Sampling strategy

Personal sampling of trichloramine and THM concentrations in air was performed during 8h day or evening shift. Our samples represented daily and frequently occurring jobs and tasks such as lifeguard, swimming teacher, baby swimming teacher, aquaerobics instructor, gym instructor, attendant, receptionist, cleaner, and administrator. The exposure time, intensity and distribution of working tasks varied considerably between the different facilities and also between different swimming pool workers. To provide detailed information, each worker kept a work diary indicating the time spent in different tasks.

Stationary air sampling of trichloramine and THM at the poolside were performed during 8h day or evening shift, as well as at locations where specific activities were performed. Samplers were placed at different fixed positions around the pool facilities for ordinary exercise or practice, at ~1.5 m above the floor level and 0–1 m from the poolside. Different locations were chosen for 8h stationary sampling: reception, exercise pool for ordinary exercise or practice for swimming clubs, training pool for swimming school, whirlpool, leisure pool, attendant office in the basement, and a gym. Stationary sampling was also carried out for a shorter period of time during specific activities such as swimming lessons, baby swimming, aquaerobics, and practice for swimming clubs. For the short term measurements during swimming lessons and baby swimming, the samplers were placed on a tripod with an extension ~0.5 m above the water surface to better represent the swimming teacher’s exposure.

### Exposure groups

Trichloramine concentrations from personal samples were classified into exposure groups based on time spent in different jobs and areas according to their work diaries (Table 2). The low exposed group spent no time in the pool area, the medium exposed spent <50% of their work shift in the pool area and the high exposed spent >50% of their workday in the pool area. The employees were considered exposed when they were working in the pool area as lifeguards, swimming teachers, baby swimming teachers, aquaerobics instructors, or gym instructor (when the gym was located in the pool area). Work tasks elsewhere in the facility were defined as low exposure jobs.

**Table 2.** Trichloramine concentrations (µg m^−3^) from personal sampling in indoor swimming pools, grouped by proportion of time spent in the swimming pool area.

Exposure group	*n*	Trichloramine (µg m^−3^)				
		AM	SD	GM	GSD	Range
Low exposed (no time in the pool area)	23	22	29	11	4	<1–110
Exposed (<50% of work shift in the pool area)	8	68	44	58	2	27–160
High exposed (>50% of work shift in the pool area)	21	120	55	110	2	48–240
Total	52	71	64	36	4	<1–240

*n*, number of samples.

### Air sampling and analysis of trichloramine

The sampling of trichloramine in air was conducted using the method proposed by Hery *et al.* (1995). Trichloramine was collected using two glass fiber filters (Whatman Grade QMA 37mm diameter) impregnated with a solution of sodium carbonate and diarsenic trioxide. We did not use Teflon filters prior to the arsenic impregnated filer in this study. This might result in that some of our measurements are affected by chloride compounds in airborne water droplets, however some preliminary tests indicated that the removal of the Teflon filters did not affect the results in our setup. The glass fiber filters were attached to a twin-port sampler (MSA Gemini®, USA) and connected to an air pump (GSA SG4000 or SKC AirChek 5000) operated at an airflow rate of 0.25 l min^−1^. The second filter was used as a back-up filter to determine a possible overload of trichloramine on the first filter. The maximum sampling time on each filter was 10h.

The purpose of the impregnation of the filter is to reduce the trichloramine collected on the filter to chloride ion (Cl^−^). After sampling, the impregnated filter was desorbed in 10ml twice distilled water and placed in an ultrasonic bath for 10min. The solution was filtered through a 13mm syringe filter (IC Acrodisc®, PALL, USA). The chlorides were analyzed in a suppressed ion chromatography system (Triatlon 900 autosampler, Spark, The Netherlands); ICSep AN1, Anion column (CETAC, Omaha, USA); SCX membrane suppressor column (Sequant, Umeå, Sweden); JD-21 conductivity detector (Costech Microanalytical Ltd, Tallin, Estonia). The eluent was 10mM NaOH with 25% acetone, 50mM H_2_SO_4_ was used as suppressor. Control samples of two known chloride concentrations (0.5 and 3mg l^−1^) and at least two blanks were run together with the samples in each run. The chloride concentrations in the blanks were subtracted from the concentrations in the samples. The limit of detection (LOD) was 0.213 µg per sample; the corresponding air concentration levels based on a flow rate of 0.25 l min^−1^ and sampling time of 1–8h ranged from 0.014 to 0.002 µg m^−3^. The analyses of trichloramine were performed in the laboratory of the Department of Occupational and Environmental Medicine at Umeå University Hospital, Sweden.

### Air sampling and analysis of THMs

The sampling and analysis of THM levels in air were based on a published EPA-method (US Environmental Protection Agency, 1999). The samples were collected on to multi-bed thermal desorption tubes (Carbotrap™ 300 Perkin Elmer, USA) connected to a twin-port sampler (MSA Gemini®, USA) and an air pump (GSA SG4000 or SKC AirChek 5000) with an air flow rate kept at 0.01 l min^−1^. The maximum sampling time on each tube was 4h; a consecutive sample was used if sampling time exceeded 4h. The samples were desorbed and injected using an automatic thermal desorption system (ATD TM, Perkin Elmer, USA) into a gas chromatograph (GC; 6890, Hewlett Packard, USA) equipped with a Rxi™-1ms column (60 m × 0.25mm and 1.0 µm film thickness, Restek, USA) and a mass spectrometry detector (MS; 5973, Agilent, USA). Helium was used as carrier gas. The samples were desorbed for 5min at 250°C with a helium gas flow at 50ml min and cryofocused at −30°C on a cold trap consisting of Tenax TA®. Finally, the cold trap was heated to 250°C and the samples split injected (outlet split 10ml min^−1^, desorb flow 50ml min^−1^) onto the GC in 4min. The temperature programme for GC was as follows: 1min at 30°C, with 5°C min^−1^ to 125°C, 15min isotherm. The identification of the four THMs were made by acquisition in single ion monitoring (SIM) mode (*m/z* for CHCl_3_/CHCl_2_Br; 82.9, 84.9, CHClBr_2_; 126.8, 128.8; and CHBr_3_ 172.8, 174.8) with the ion source operating at 230°C and 70eV. Integration and evaluation of the chromatograms and spectrums were made using computer software (G1701DA MSD ChemStation Version D.00.00.38, Agilent tech, USA). Our lowest concentration levels were well above the limit of quantification (LOQ); signal to noise ratio (S/N) larger than 10 except for CHCl_2_Br (S/N 4.7). The LOQ was 0.75ng per sample (CHCl_3_) and 0.10ng per sample (CHCl_2_Br, CHClBr_2_, and CHBr_3_). A flow rate of 0.01 l min^−1^ and a sampling time of 4h resulted in corresponding air concentrations of 0.3 µg m^−3^ (CHCl_3_) and 0.04 µg m^−13^ (CHCl_2_Br, CHClBr_2_, and CHBr_3_). The analyses for THM were performed in the laboratory of the Department of Occupational and Environmental Medicine at Örebro University Hospital, Sweden. The Swedish Board of Accreditation and Conformity Assessment (SWEDAC) has accredited the laboratory at the Department of Occupational and Environmental Medicine in Örebro.

### Statistical methods

For descriptive purposes arithmetic mean (AD), standard derivation (SD), geometric mean (GM), geometric standard deviation (GSD), and range were calculated for the trichloramine and THM concentrations. Data from personal samples of trichloramine were presented for different groups: low, medium, and high exposed, based on time spent in the swimming pool area. Stationary measurements were presented for different locations and activities in the facilities. Personal and stationary measurements of THMs were summarized for all facilities. The LOD was defined as a signal-to-noise ratio of 3 and the LOQ was defined as a signal-to-noise ratio of 10 (EURACHEM, 1993). For air concentrations of trichloramine below the LOD, LOD/√2 was used as data for calculations. For air concentrations of THM below the LOQ, LOQ/√2 was used as data for calculations (Hornung and Reed, 1990).

For linear regression analysis and prediction of personal exposure to trichloramine from stationary sampling, data from parallel personal and stationary sampling during 8-h measurements around exercise and training pools were used. Only samplings from personnel who spent > 50% of their workday in the pool area (the high exposed group in Table 2) were included. The comparison was made between arithmetic mean from personal sampling (if more than one personal sampling was conducted at the same time) and arithmetic mean from stationary samples that were connected with the personal samples. In some of the pool facilities more than one such parallel measurement was made, one for the day shift and one for the evening shift. These 12 different parallel measurements were used for the linear regression. The Statistical Package for Social Sciences (SPSS) for Windows 22.0 was used for all calculations.

## RESULTS

### Trichloramine

#### Personal sampling

In total, 52 personal samples of trichloramine were collected. The sampling time ranged from 2 to 10h, 75% of the samples had a sampling time longer than 6h. The trichloramine concentrations for the personal sampling varied between <1 and 240 µg m^−3^ and the GM was 36 µg m^−3^. The trichloramine concentrations for the low exposed group varied between <1 and 110 µg m^−3^and a GM of 11 µg m^−3^. For the high exposed group, the concentrations ranged from 48 to 240 µg m^−3^; GM was 110 µg m^−3^ (Table 2). The highest trichloramine concentrations (220 and 240 µg m^−3^) were measured for a lifeguard and a swimming school teacher.

#### Stationary sampling

In total, 110 stationary samples of trichloramine were collected. The trichloramine concentrations for stationary samples, representing locations and specific activities, varied between <1 and 640 µg m^−3^ and a GM of 100 µg m^−3^. The levels at the receptions were low, ranging from <1 to 40 µg m^−3^. The AM for exercise, training, whirl- and leisure pools ranged from 150 to 370 µg m^−3^, with corresponding overall range from 50 to 540 µg m^−3^. One stationary sample near a whirlpool (540 µg m^−3^)and one taken during a water aerobics session (640 µg m^−3^) exceeded the recommended WHO-reference value for trichloramine (500 µg m^−3^) (Table 3).

**Table 3.** Trichloramine concentrations (µg m^−3^) from stationary sampling in indoor swimming pools, grouped by location and activity.

Stationary sampling	*n*	Trichloramine (µg m^−3^)				
		AM	SD	GM	GSD	Range
Reception	15	7	10	4	3	<1–40
Exercise pool	39	150	76	140	2	50–400
Training pool	10	170	61	170	1	100–290
Whirlpool	7	370	120	360	1	250–540
Leisure pool	4	270	21	270	1	240–300
Swimming school	17	190	90	170	2	69–400
Baby swimming	7	190	130	160	2	88–460
Aquaerobic	4	390	190	350	2	180–640
Practice for swimming clubs	5	280	88	270	1	160–400
Attendant office in basement	1	24				
Gym	1	200				
Total	110	170	130	100	4	<1–640

*n*, number of samples.

#### Regression and prediction

**Figure 1.**
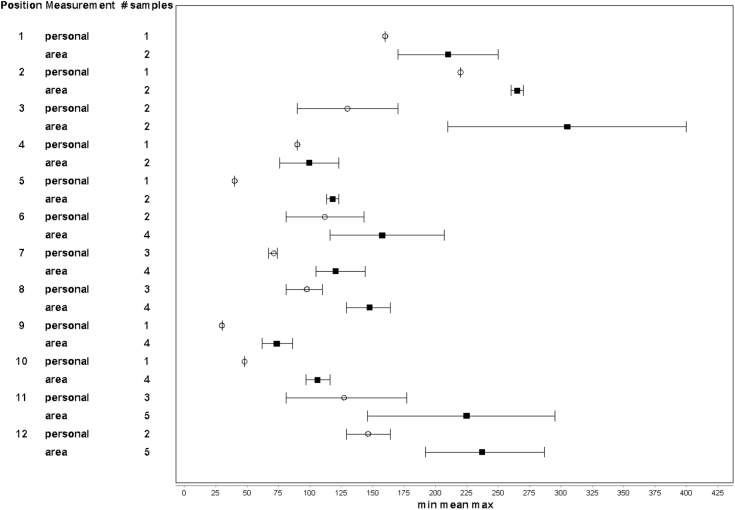
The arithmetic mean and range for the 12 different parallel measurements (area ▪ and personal ◦) of trichloramine for the personnel working >50% of their work-shift in the swimming pool area (the high exposed group).

The AM of the 12 different parallel measurements (area and personal) of trichloramine was calculated (Fig. 1). Linear regression analysis for trichloramine levels from the exposure measurement data to the corresponding stationary measurement at the pool side showed a correlation coefficient (*r* ^2^) of 0.693, indicating ~70% explained variance and a significant regression coefficient (*β* = 0.621; 95% CI = 0.329–0.912, *P* = 0.001). A Bland Altman test of the material gave a mean difference between personal and stationary measurement of −66 µg m^−3^ (95% CI = −147 to 16).

Regression analysis reflects the relationship between mean of stationary measurements and mean of the exposure measurements (y_(pred)_ = −0,71 + 0,62x). Predicted individual exposure concentrations from stationary concentrations of trichloramine and corresponding 95% CIs for stationary trichloramine levels of 50, 250, and 500 µg m^−3^ are shown in Table 4. The predicted individual concentrations were 30 µg m^−3^ (95% CI = −11 to 71), 154 µg m^−3^ (95% CI = 124–185) and 310 µg m^−3^ (95% CI = 212–407), respectively (Table 4).

**Table 4.** Linear regression analysis by trichloramine concentrations (µg m^−3^) from stationary sampling in indoor swimming pools as independent variable and personal sampling as dependent variable (*n* = 12).

Agent	Regression				Prediction			
	*β*	*P*-value	95% CI (*β*)	*r* ^2^		Assumed concentration	Predicted concentration	95% CI (*y*)
Trichloramine	0.621	<0.001	0.329–0.912	0.693		50	30	−11 to 71
						250	154	124 to 185
						500	310	212 to 407

95% CI (*β*), 95% confidence interval for *β*; *r* ^2^, correlation coefficient; predicted concentration, predicted individual concentration for trichloramine 50, 250, and 500 µg m^−3^; 95% CI (*y*) = 95% confidence interval for predicted dependent metrics, individual concentrations.

### Trihalomethanes

#### Personal and stationary sampling

In total, 52 personal samples and 110 stationary samples of THMs were collected in parallel with both the exposure and stationary measurements of trichloramine. Two of the THM samples were excluded due to water contamination leaving 51 personal and 109 stationary for analysis. CHCl_3_ was the dominating THM in the personal samples and varied between 3.0 and 170 µg m^−3^. The other THMs showed concentrations of <0.04–19 µg m^−3^ (Table 5). CHCl_3_ was also the dominating THM at the stationary measurements with concentrations between 0.13 and 220 µg m^−3^. Concentrations at stationary sampling of the other THMs varied between <0.03 and 21 µg m^−3^ (Table 5). The highest CHCl_3_ concentration at personal sampling (170 µg m^−3^) was measured on a employee teaching swimming school and working as a lifeguard in an exposed environment. The highest recorded stationary measurement of CHCl_3_ (220 µg m^−3^) was measured at the exercise pool in the smallest facility. Both personal and stationary samples of CHCl_3_ were well below the Swedish OEL of 10000 µg m^−3^. A linear regression analysis for CHCl_3_ on the personal exposure measurement to the corresponding stationary measurement at the pool side gave a significant regression coefficient (*β* = 0.560; 95% CI = 0.098–1.022, *P* = 0.022). Regression analyses where not performed on the other THMs as the measured levels of these where low.

**Table 5.** THM concentrations (µg m^−3^) from personal and stationary sampling in indoor swimming pools.

Chemical agents	*n*_tot_	Air concentrations (µg m^−3^)				
		AM	SD	GM	GSD	Range
CHCl_3_						
Personal	51	30	31	19	2.7	3.0–170
Stationary	109	54	49	28	4,2	0.13–220
CHCl_2_Br						
Personal	51	3.9	4.2	2.4	2.9	0.21–19
Stationary	109	5.5	4.8	3.3	3.4	0.08–21
CHClBr_2_						
Personal	51	0.99	1.6	0.43	3.2	<0.04–8.3
Stationary	109	1.4	2.2	0.54	4.2	<004-14
CHBr_3_						
Personal	51	0.14	0.18	0.09	2.5	<0.04–0.99
Stationary	109	0.17	0.40	0.06	3.4	<0.03–3.6

*n*, number of samples.

CHCl_3_ concentrations where also found to be correlated with trichloramine (*β* = 0.084; 95% CI = 0.011–0.157, *P* = 0.02), however the *r* ^2^ was low, 0.047.

## DISCUSSION

To our knowledge this is the first study that has measured exposure to chloramines simultaneously by both personal and stationary sampling within indoor swimming pools. It has long been known that measurements at fixed locations within a work environment may not reflect personal exposure (Sherwood and Greenhalgh, 1960). Most previous occupational studies did not find any relation (Stevens, 1969; Linch and Pfaff, 1971; Lange, 1999; Lange and Thomulka, 2000; Lange *et al.*, 2000; Linch *et al.*, 1970), but some did (Breslin *et al.* 1967; Lange *et al.* 1996), between personal and stationary sample measurements. Personal samples are generally higher in concentration than stationary samples because of people being closer to the source and spending more time within the source location, or in the emission pathway (Leung and Harrison, 1998; Lange *et al.*, 2000). It has been found that personal exposure measurements can be 1.2–8.5 times higher than simultaneously collected fixed location samples (Botham *et al.*, 1988; Cherrie and Stelfox, 1990; Sahle *et al.*, 1990; Cherrie *et al.*, 1991, 1995; Ogden *et al.*, 1993; Cherrie and Schneider, 1999). However, if stationary samplers are placed at the source location or emission pathway, they are similar to the values reported for personal samples (Corn, 1983; Lange, 1999) and, in some incidents, may show a higher concentration (Lange et al., 1996, 2000; Lange and Thomulka, 2002). There are some factors that are crucial to determine the relation between personal and static measurements: the volume of the room, the quantity of general ventilation, the time the person spends near the sources of hazardous substances and the presence of other sources of the contaminants (Cherrie, 2004). The difference between personal and stationary sampling seems to be lower during poor ventilation conditions (Cherrie, 2003).

In this study, extensive personal and stationary measurements of trichloramine and THMs were performed at eight Swedish swimming pool facilities during winter seasons from January 2007 until March 2009. Our personal samples represented pool workers’ daily activities which often varied both within and between various locations in the pool facilities. Stationary samples were located along the pool sides and also reflected different types of activities, all sampled in parallel to the personal measurements.

The range of measured trichloramine concentrations was similar or lower than those found in previous studies. Air concentrations of trichloramine in indoor swimming pool facilities in a French, British, Dutch, and Swiss study varied between 20 and 1340 µg m^−3^ (Massin *et al.*, 1998; Thickett *et al.*, 2002; Jacobs *et al.*, 2007; Parrat *et al.*, 2012).

None of the measured personal trichloramine levels (*n* = 52) exceeded 250 µg m^−3^ and all were well below the recommended WHO reference value of 500 µg m^−3^. The two highest values (220 and 240 µg m^−3^) were measurements from workers that, according to their work diaries, mainly worked in an exposed environment as a lifeguard and as a swimming school teacher.

Two of the stationary samples of trichloramine (*n* = 110), one sample from an aquaerobics session (640 µg m^−3^) and another stationary sample situated near a whirlpool (540 µg m^−3^), exceeded the recommended WHO-reference value for trichloramine (500 µg m^−3^). This is in agreement with previous studies that found higher levels of trichloramine in air during vigorous water movements (Hery *et al.*, 1995; Massin *et al.*, 1998; Thickett *et al.*, 2002). The same pattern with higher trichloramine concentrations was seen at stationary measurements close to whirlpools, the leisure pool, aquaerobics sessions, and swimming practices. As expected, low air concentrations of trichloramine were registered in the reception areas and in the basement. More diverse trichloramine concentrations were found at stationary measurements nearby exercise and training pools and during swimming school and baby swimming sessions.

The reference value from WHO is based on stationary measurements from a French study (Hery *et al.*, 1995), and a Swiss study that suggests a trichloramine OEL at 300 µg m^−3^ is also based on stationary measurements (Parrat *et al.*, 2012). Levels of trichloramine found in this study, however, suggest that exposure measurements from high exposed workers have approximately half as high concentrations compared with those measured in parallel stationary measurements (Table 4). With these assumptions, we suggest that if stationary exposure measurements are used as a base for an OEL, this exposure limit should be half as high when used for personal exposure.

Exposure measurements of THM are, as with trichloramine measurements, rare. The majority of published studies mainly include evaluation of swimmers with respect to the different pathways of THM-uptake or the THM-concentrations in their blood. The THM-concentrations found in this study (0.13–220 µg m^−3^) were of the same order, or much lower, compared to other studies. Air concentrations of CHCl_3_ in indoor swimming pool facilities in three German and five Italian studies varied between 10 and 853 µg m^−3^ (Lahl *et al.*, 1981; Aggazzotti *et al.*, 1990, 1993, 1995, 1998). The CHCl_3_ concentrations in air found in this study were far below the Swedish OEL (10mg m^−3^). Despite the absence of OELs for the other THMs (CHCl_2_Br, CHClBr_2_, and CHBr_3_) we conclude that the air concentrations in this study are low.

## CONCLUSIONS

Most of the personal air concentrations of THM and trichloramine measured in this study were low. However the results in this study suggest that high exposed workers have approximately half as high exposure to trichloramine compared with stationary measurements performed in parallel. This should be taken into consideration when setting an OEL for trichloramine, as most of the published studies on health effects from occupational trichloramine exposure in swimming pools have used stationary measurements for exposure assessment.

## FUNDING

This study has been financed by the county councils of Örebro, Västmanland, Värmland, and Södermanland.
